# When the self “*logs in*” - a critical narrative review of digital identity in health professions education

**DOI:** 10.3389/fmed.2025.1715752

**Published:** 2025-12-18

**Authors:** Shaista Salman Guraya, Farah Ennab, Salman Yousuf Guraya

**Affiliations:** 1 Institute of Learning, Mohammed Bin Rashid University of Medicine and Health Sciences, Dubai Health, Dubai, United Arab Emirates; 2 College of Medicine, Gulf Medical University, Ajman, United Arab Emirates

**Keywords:** digital identity, digital platforms, socio-material theory, impression management, narrative self, digital agency, health professions education, symbolic interactiosnism

## Abstract

Digital identity is no longer an add-on to professional life; it is a primary arena where the self is performed, negotiated, and sustained. Health professions education (HPE) depends on visibility; learners seeking mentors, academics signaling scholarship, clinicians building legibility on rating sites. Yet that same visibility is cross-pressured by codes of conduct, context collapse, and the ethics of self-disclosure. This critical narrative review treats digital identity as identity-as-work in public, persistent, searchable systems (the sum of traces others later encounter when platforms remix and rank them). Bringing symbolic interactionism (performance, audience, impression management) into conversation with a sociomaterial stance (platforms and artifacts co-produce action), and drawing a light Lacanian inflection where helpful, we read the corpus through four facets: authenticity, visibility, continuity (idem/ipse), and agency. Searches across Scopus, Embase, PubMed, PsycINFO, and Google Scholar returned 1,638 records; after 256 duplicates, 1,382 titles/abstracts were screened, 234 full texts assessed, and 45 sources included. Reflexive memos and iterative comparison guided an interpretive synthesis discussed with wider team. Two recurrent modes (tropes) surfaced. In a mediating mode, “digital neurotic self” curates authorship under constraint, running legibility tests (Is this true to my values? Which audience will see it? What trace will it leave?) and adjusting voice, timing, and placement. Practices include audience design (lists, close-friends, pseudonyms), contextual disclosure, dual-account compartmentalization, and portfolio stitching to maintain continuity while staying findable. In an instrumentalised mode, the ‘digital psychotic self’ is built for consumption and tuned to platform legibility; counters, templates, and recommendations, thin authored selfhood, nudge toward micro-celebrity, and drift from ipse (authored) to idem (community sameness). Across studies, homophily, metrics, and templated formats push toward uniformity; without active curation, narrative coherence frays into platform-tuned fragments. For HPE, we argue that digital identity is a dimension of professional identity formation. Educators should coach authorship and stewardship (audience design, narrative/portfolio stitching, sociolinguistic competence), teach platform literacy (how feeds rank/normalize; how ratings/altmetrics discipline presentation), and create counter-spaces that protect backstage rehearsal while enabling intentional visibility. Finally, help learners move beyond perpetual exploration toward value-anchored commitments, so visibility becomes a record of work rather than a performance for counters.

## 1 Introduction

Digital identity is no longer an add-on to our social lives (1). It has become an integral arena for performing, negotiating, and sustaining the self (1, 2). This rapid yet almost imperceptible migration of identity work into digital spaces has unsettled established social norms and values, raising urgent questions about authenticity, visibility, and autonomy (3, 4). In today’s networked society, digital identity has become part of everyday work, our social presence and professional life intertwine, demanding conscious choices about openness, multiplicity, and reputation (3). Scholarship on digital identity tends to fall into two broad camps: one treats it as a matter of personal expression shaped by psychological and social influences (5) while the other frames it as a product of platform architectures and algorithmic logics (6). Yet, these perspectives rarely integrate the symbolic processes of meaning-making with the material infrastructures that both enable and constrain them. Beyond everyday “profile building,” much of the literature treats digital identity as a reconstruction, selective, ideal, or even false self-presentation to meet social, security, or exploratory needs, framing our focus on identity-as-work in socio-technical systems (5).

Historically, our sense of self was anchored in the family, local communities, (proto)ethnic groups or tribes, and religious groups. Identities were formed in physical spaces, allowing people to feel grounded and consistent even as circumstances and settings shifted. As contexts have transformed from the physical to the digital, identity has evolved into a fluid and multifaceted digital identity, a carefully constructed expression of self that operates across personal, social, and professional domains (7). Social media platforms now serve not only as online social spaces but also as stages where professional identities are constructed, negotiated, and performed (8–10). This identity choreography highlights a double edge: digital platforms empower visibility and connectivity, yet the same architectures can invite conformity, temper idiosyncrasy, and reshape how uniqueness is signaled and recognized (5).

For healthcare professionals (HCPs), identity work increasingly unfolds in public, persistent, searchable spaces where platform logics shape what is sayable and seen (11). Health professions education (HPE) depends on visibility; learners seeking mentors and audiences, academics signaling scholarship (12, 13), clinicians building trust and legibility in a world of patient-rating sites (14). Yet, that same visibility is cross-pressured by professionalism and codes of conduct, context collapse, and the ethics of self-disclosure (9). Early-career “digital natives” often want to make small wins permanent online; established clinicians (15, 16) feel the pull to protect reputation, reassure patients, and uphold organizational standing. HCPs should assume hiring and promotion committees will Google them; page-one results (institutional bio, LinkedIn/ORCID/Scholar, patient rating websites) shape trust and employment outcomes, so the curated digital trace becomes part of the dossier (17–19). Yet, across roles, the everyday decision is the same: “*to post or not to post*” how to be findable and credible without being flattened by metrics or exposed to harassment (20–25). HPE scholarship already shows that a digital component of professional identity forms to meet role needs, develops in parallel with offline identity, and therefore cannot be treated as “*extra*,” it is part of professional identity work (26) as deliberate curation, not just expression (27). However, much of the HPE literature still treats online presence as either “branding” or policy compliance, under-theorizing identity work itself.

We treat digital identity as the ongoing negotiation between a “*substantive self*” (anchored in self-defining beliefs and values) and a “*situational self*” (anchored in context and the expectations of audiences and platforms) (28). To fully understand this rhetoric, we draw on two complementary perspectives. Drawing from the sociological tradition of symbolic interactionism, where identity is understood as a dynamic and performative social construction, we recognize that identity work has historically centered on human interactions and shared meaning-making (29). Unlike purely symbolic or agentic accounts of identity, sociomaterial theory foregrounds the co-constitutive relationship between human subjects and the digital infrastructures that shape their visibility, expression, and behavior (30). In this review article, we adopt sociomaterial theory (10, 30) to explore how digital platforms do not merely provide the backdrop for this identity performance but act as active participants in shaping how identities are enacted, curated, and sustained. The materiality of these platforms, their algorithms, visibility metrics, and content affordances interacts with our social behaviors, co-producing a dynamic environment in which the self is continuously reassembled.

This realization urges us to confront a set of critical provocations rather than a single delimiting research question. How are digital platforms reshaping and, in many cases, eroding our sense of unique identity? Is self-presentation an authentic expression or a constructed illusion? What does it mean to “be someone” in an environment where platforms continuously measure and influence visibility, behavior, and even values? Situated within an interpretive paradigm (31), this critical narrative review adopts these critical interrogative provocations as dialogic reflective entry points in an allegorical structure that mirrors the iterative, fluid, contested and conversational nature of identity formation itself.

## 2 Methodology

We searched PubMed, Scopus, Embase, PsycINFO, and Google Scholar using a single key term as an exact phrase “*digital identity*” with database-specific automatic term mapping where available. No synonyms or controlled vocabulary expansions were used to keep the scope deliberately narrow and centered on identity as “*self*” rather than authentication technologies. Limits were publication in English and in a time frame from 2000 to 2025. For Google Scholar, we applied a reproducible stopping rule (custom date range; screening of the first 10 pages of results, and cessation when consecutive pages produced no new concepts). Reference lists of included records and their forward citations were hand-searched to identify additional sources.

We included publications that explicitly examined digital identity as identity work (e.g., online persona, self-presentation, narrative identity, e-professionalism, digital footprint) on social/digital platforms or e-portfolios; were relevant to HPE or adjacent higher-education/professional practice with clear transferability to HPE; and engaged at least one sensitizing lens pertinent to identity (e.g., symbolic interactionism/dramaturgy; sociomateriality of platforms/affordances/algorithms; narrative identity/continuity). Eligible empirical and non-empirical research included qualitative, mixed-methods, concept analyses, theory papers, and critical essays offering definitional, mechanistic, or conceptual contributions. We excluded records focused solely on technical identity management (e.g., eID/digital ID wallets, KYC, SSO, OAuth, blockchain credentials) or cybersecurity/biometrics without identity-as-self analysis; corporate brand/marketing persona without individual identity work; patient identity verification and record-matching in health systems; opinion/editorials that contributed no new definitions, mechanisms, or empirically anchored insights; and non-English items or records without retrievable full text.

Records were exported from databases and managed in Covidence for de-duplication, screening, and documentation. Screening proceeded in two stages against the pre-specified criteria: title/abstract screening followed by full-text assessment. Hand search citation chasing was conducted from included items until conceptual saturation (no new concepts emerging over successive rounds). For each included item, we extracted: bibliographic details; discipline/context; definitions or typifications of digital identity; attention to platform materiality, identity work facets, population and professional stage; salient metaphors and quotations; and implications for HPE (learners, educators, and organizations). Extraction was recorded in structured matrices to support transparency and synthesis.

Consistent with a critical narrative review and in line with the Scale for the Assessment of Narrative Review Articles (SANRA) procedure (32), we prioritized conceptual contribution over risk-of-bias scoring. Each item was appraised on three dimensions: (1) relevance to our lenses (identity work and platform materiality), (2) attention to mechanisms, meaning how affordances and metrics shape identity, and (3) transferability to HPE practice. Lower-contribution pieces were retained only for background or definitional context.

### 2.1 Reflexivity

As researchers, our positionalities, shaped by our medical backgrounds, life experiences, and personal beliefs, inevitably shaping the way we approached and interpreted the concept of digital identity. For instance, I (SSG), who has studied both professional identity creation and digital cultures, approached this question from an academic and a practical point of view. I acknowledge that the claims made here emerge from my immersive experiences in the research of e-professionalism, digital environments, and I heavily rely on Denzin’s interpretive traditions in qualitative research (31). My view sits at the intersection of multiple disciplines and is grounded in the belief that theory is a tool for critical sense-making rather than distant abstraction. Additionally, the diverse positionalities of my co-authors also shape this paper. One senior author (SYG), a surgeon coming from a post-positivist world, holds a firm belief in the mattering of matter within the sociometrical theory. He emphasizes the importance of a strong digital presence. He intentionally curates and creates content to project an academic and researcher identity. Among us, FE is the Gen-Z collaborator, a strategic content creator who understands how to engage and captivate audiences, aiming not for sheer follower counts but drawing deeply from the aesthetic and stylistic influences of those who inspire her. She is currently pursuing her MSc HPE and is challenging the disciplinary discourse by rooting for indigenous and native educator identity formation.

Operationally, SSG conducted the searching, screening, extraction, and initial synthesis. Following the preliminary synthesis, interpretations were discussed with the wider team through structured peer-debriefing (SYG, and FE) to surface assumptions, consider rival explanations, and test theme fit against counter-examples. Throughout, we maintained reflexive memos to make interpretive moves and value judgments explicit.

### 2.2 Analytical approach

Given the interpretive nature of this critical narrative review, the analysis was not conducted through coding or evidence extraction but through theoretically guided reading. Goffman’s dramaturgy (frontstage/backstage; audience-shaped performance) inspired symbolic interactionism (10, 30, 33), and sociomaterial stance (people–technology co-production; platforms materialize norms, govern visibility, incentivize performances) (34), which renders identity post-structural dispersed across sites, something to curate rather than possess directed our attention respectively to performance, audience, impression management, and to platform affordances, metrics, and visibility served as the two primary analytic lenses that organized how the literature was approached. As the reading progressed, the literature also invited a Lacanian inflection of extimacy, the feeling that the “*outside*” (feeds, analytics) sits inside the self, which helps explain why metrics can mirror and discipline us so closely (4, 7). These lenses shaped the development of our conceptual tropes, as described in the results section, through an iterative movement between the literature and the theoretical frames, asking how each text illuminated or unsettled assumptions about identity, coherence, visibility, and platform-mediated selfhood. The analysis, therefore, represents a theory-led synthesis rather than a systematic classification.

For the sake of analysis, we treat digital identity as the sum of traces we leave online, willingly or not, and the composite others later encounter once those traces are remixed and ranked by search engines and social networks. “Authenticity” here refers to the fit between a public profile and lived experience; many accounts assume that an offline identity is “*genuine*” and an online identity is a “*curated*” (or even corrupted) rendition (29, 35). This keeps our analysis on *identity-as-work* in public, persistent, and searchable domains. We begin by tracing philosophical and psychological traditions that have long underwritten ideas of the self. Thinkers like Locke and Kant spoke of “*self-persistence*” or “*self-sameness*” (36), and Erikson explored psychological coherence across time as continuity, stability, and agency (37); qualities that, in our reading, contemporary platforms fragment and reconfigure (7, 8). As we read further, a Lacanian inflection helped to name what we were seeing. Extimacy captures the paradox that what appears most external to us, technical systems and networked publics becomes *intimately constitutive* of inner life, sometimes read (problematically) as “*technological determinism*” (7). For our purposes, extimacy simply foregrounds how digital and algorithmic environments are embedded in subjectivity: they help shape desire, identity, and relations in ways that are not always transparent yet without fully determining them. There remains a cavity, an internal gap that preserves the possibility of singular self-formation (4, 7). Turning to sociomaterial theory, we then asked how the self is formed not only through interaction with people but also through platforms that materialize social norms, govern visibility, and algorithmically incentivize particular performances. On this view, digital identity appears poststructural, aggregated and dispersed across sites, with multiple presences in perpetual flux, something to curate rather than possess (33). This dialogic movement between the symbolic and the material set the scene for our synthesis.

Within the corpus, one source described digital identity as communicative presence “*who you are/want to be*” realized in language and read by the community built from epistemic views and social, cultural, and linguistic capital, with the community reciprocally interpreting and positioning each contribution (34). In practice, what derails differs by mode: in a mediating mode, our “*digital neurotic self*” (metaphorical), these capitals are anxiously calibrated to keep idem (public sameness) aligned with ipse (authored selfhood). While in an instrumentalised mode, our “*digital psychotic self*” (metaphorical), platform taxonomies and data-mining logics recode those capitals into engagement-legibility, bending desire toward algorithmic categories and thinning authored selfhood. We therefore read the corpus with a dual focus on performance (audience, impression management) and materiality (affordances, metrics, governance), and then asked what patterns this combination reveals (7).

Read through an ipse/idem lens, studies by Xu and Jing (38), Ndumu and Onianwah (39) underline how platforms quietly run on homophily: we affiliate when “*identity meanings*” align, producing a felt “*we-ness*” that converges into homogeneous personae (40). In that drift toward community sameness (idem), the authored ipse is easily thinned into platform-readable uniformity unless actively curated. Classic impression management (33) helps here by stating interaction as a performance. We act frontstage for audiences and adjust backstage out of view. Online, the stage is built by affordances, metrics, and norms, so performance is shaped as much by interfaces as by people. The digital selfhood sits between audience-defined presentation and reflexively authored identity. Few reach a fully relational self, and the majority were depicted as Kegan’s fifth-order capacities (holding multiple frames and working on the self) (8). The literature accordingly describes an increasingly generalized publicisation of the self. From a sociomaterial angle, “*influence*” is not backstage magic; it emerges when creators’ practices are co-authored by recommendation engines, counters, and brand-safety rules. In our synthesis, these dynamics repeatedly resolved into two patterns: a mediating mode in which people test for legibility (values, audience, trace) and tune voice, timing, and placement to stay authentic while visible; and an instrumentalised mode in which performance is optimized for what travels, keyed to counters, templates, and trends rather than to meaning (39). Reading the corpus, Haltigan et al. (41) especially nudged us to see two modes: a cautious, mediated self versus an instrumentalised, metrics-led self, showing how algorithmic audiovisually immersive feeds can incubate identity performances and even social contagion, highlighting that platforms don’t just host feelings, they can steer them, shifting regulation from within the self to through the feed. Additionally, reading Holmes (42) on Serial Experiments Lain nudged our “*digital psychotic self*” (metaphor): the show’s digital double, the blur between the Wired and the “*real*,” and the empathy problem born of competing drives for privacy and visibility all point to a self tuned for legibility rather than lived coherence.

Empirically, across studies, the online self often appears as scattered, platform-tuned fragments that leak across time and audiences; without active curation, the “*life story*” frays, and what others meet is a collage rather than a coherent self (43). Coherence is re-stitched when traces are remediated (e.g., assembled into portfolios or longer-form narratives), suggesting that fluid, extended presences easily outgrow narrative continuity. Shi, Lai (27) operationalize platform materiality in traceable metrics (followers, post length, retweet interval, bandwagon counts) and show that people often stay within familiar domains to signal a coherent self, classic cautious authorship, yet the same mechanics can tip into performance-for-reach as audience size and bandwagon cues steer optimization to counters. Finally, several studies show digital affordances holding some learners in prolonged exploration, providing endless options, creating reflection loops without commitments, and a perpetual moratorium (6). Together, these strands informed rather than merely illustrated the two interpretive tropes we develop in the next section.

## 3 Results

**FIGURE 1:**
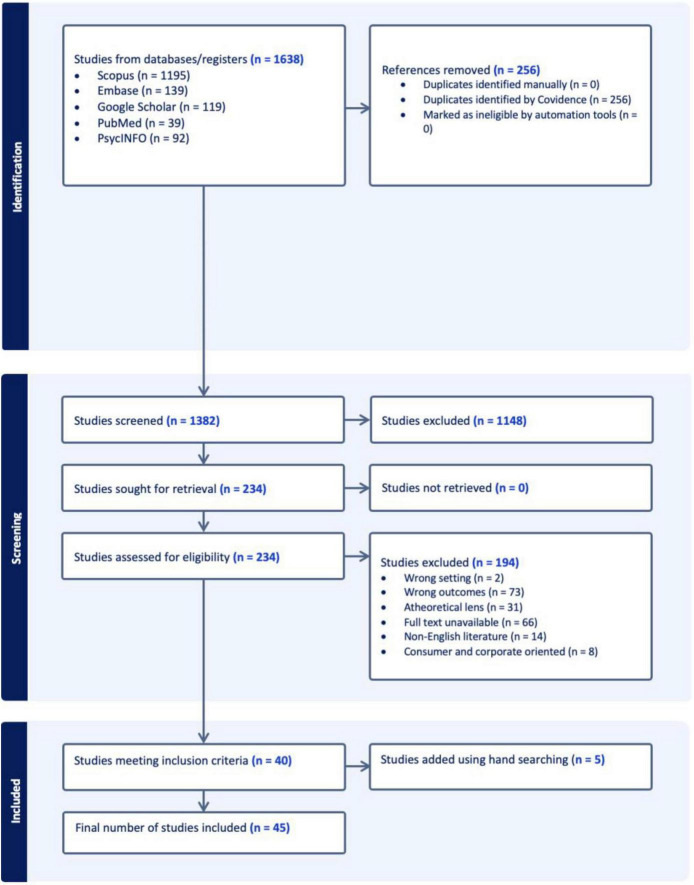
Flow diagram illustrating the literature search and theory-informed inclusion process for this review.

The search yielded 1,638 records. Scopus 1,195, Embase 139, Google Scholar 119, PubMed 93, and PsycINFO 92. After removing 256 duplicates, 1,382 titles/abstracts were screened. Subsequently, 234 full texts were assessed. A total of 45 studies were included; 40 met the inclusion criteria, and 5 were identified through hand searching (see Figure 1).

This review identified several conceptual tensions within how digital identity is theorized across the literature. The literature informing this review reflects four broad intellectual terrains: (1) sociological and symbolic interactionist writing on identity performance; (2) psychological and behavioral studies examining self-presentation and online impression management; (3) digital technology and surveillance scholarship analyzing platform power, metrics, and visibility; and (4) empirical work documenting lived experiences of digital identity among professionals from various walks of life and youth. Across these terrains, methodological diversity is the norm, encompassing conceptual essays, qualitative studies, mixed-methods analyses, and theoretical position papers, which highlight their valuable insights rather than the methods employed. These categories served as interpretive anchors instead of formal evidence-mapping tools, consistent with the narrative, critical orientation of the review.

**FIGURE 2:**
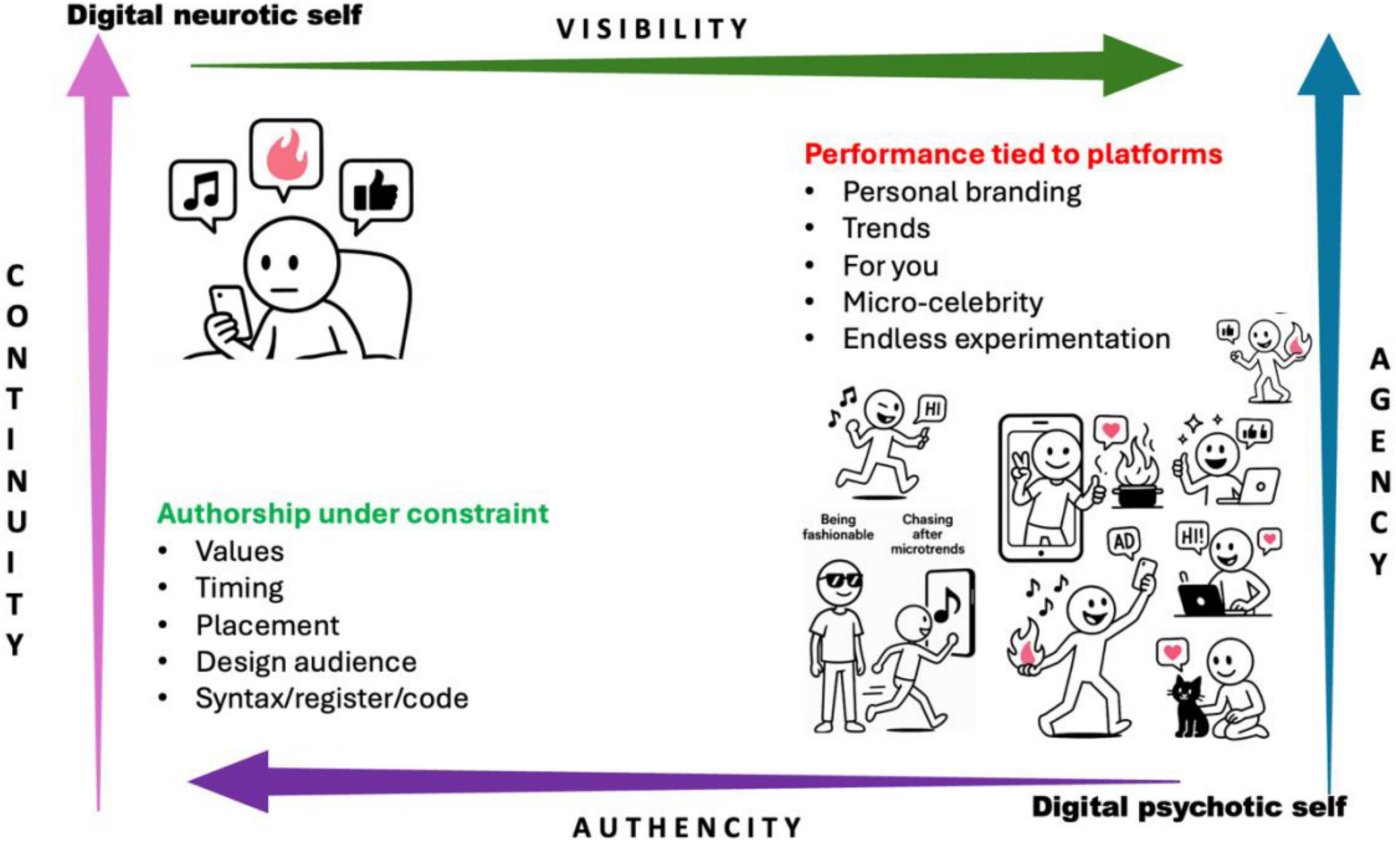
Illustration of two digital identity modes (tropes): the “digital neurotic self” vs. the “digital psychotic self.” Illustrations created using Microsoft Copilot 3.2 premium software.

Read across HPE and adjacent literatures, two recurring modes (tropes) of identity work surfaced. In a mediating mode – “*digital neurotic self*,” individuals curate authorship under constraint by testing posts against values, audiences, and context to remain authentic yet visible. In an instrumentalised mode – “*digital psychotic self*” presentation is constructed for consumption, tuned to platform legibility and norms (please note that these are in a metaphorical sense and not in clinical terms). Across the corpus, visibility pressures were most explicit in the form of searchability, audience design, counters/recommendation, authenticity was negotiated through voice and boundary work, continuity appeared where timelines and archives stitched narratives over time (idem/ipse), and agency varied with material conditions in the form of affordances, defaults, moderation and ranking often rendering identity co-authored with material interfaces (10). We present the mediating–instrumentalised heuristic as an emergent synthesis from the included studies rather than *a priori* frame consistent with an interpretive paradigmatic view (31) (see Figure 2).

### 3.1 Trope I: mediating mode — the “*digital neurotic self*” (metaphorical)

The digital world invites two distinct modes of identity performance. The first is a digital neurotic self an ego that works to preserve self-authorship by mediating between inner authenticity and the outer demand for visibility (44). It assumes a self that has wrestled with the “*Who am I?*” *and* “*Why am I?*” from personal and social reference points (10). Because identity is contextual and relational, it requires ongoing maintenance and reconstruction (45). Authenticity is negotiated, visibility is managed, continuity is stitched, and agency emerges often via self-censorship and calibration (28). Developmentally, people move from external definition (beholden to others’ views) toward internal definition (conscious, value-anchored choices about social media), signaling potential for ipse (authored selfhood) without evidence of a fully postmodern/relational endpoint (8). Think of the medical student who curates carefully to remain professional yet feels uneasy about leaving parts of the self unexpressed (46).

Across HPE and adjacent studies, identity work is curated under constraint. People post with one hand on purpose and the other on the guardrail: before publishing, they run legibility tests; “*Is this true to my values? Which audience will see it? What trace will it leave?*” and then adjust voice, timing, placement. In practice, that means audience construction (curated lists, private groups, privacy toggles, composing for actual and imagined audiences). Some adopt representative/role-model stances or write to a future self (47), treating posts as a controlled archive that preserves authenticity and continuity while staying visible. Materially, mediation is enacted through affordances, including close friend lists, account separation/pseudonyms, comment filters, hashtags for intended publics, and local norms/moderation. Integrated findings show Gen-Z using dual accounts, audience segmentation, and contextual self-disclosure often with greater disclosure and self-promotion on second accounts to reconcile authenticity and visibility under context collapse (2). Studies depict social networks as a blurring stage where “*accessible privacy*” and mediated intimacy enable authorship under constraint rather than unfiltered exposure (1). Relatedly, young users adopt a “*virtual mask*” (35) in the form of anonymity or nicknames, limited disclosure, tight audience control to avoid stigma, harassment, or punishment. Reading Kavakci and Kraeplin (48) sharpened this view of the digital neurotic self. Marginalized hijabi influencers author visibility under constraint through styling, captions, and audience curation to reconcile piety and fashion, making religious and cultural capital legible while guarding the substantive self.

Agency shows up in micro moves; draft - pause - post cycles, selective disclosure, audience design, platform choice, pacing, and time-away to manage emotional load (2). A perfect example of visibility under constraint was depicted in a qualitative study of Igbo women fashion designers on Instagram, identity work was enacted through platform affordances (reels, hashtags, DMs) and moderation constraints (algorithmic preference, shadow-banning/bias); participants used audience segmentation, stylistic coding, and situated captions to keep cultural/religious signals legible while remaining market-visible (49). This strategic self-presentation is true but curated, thus appears as a value-anchored selection under audience and platform pressure, an anxious yet coherent stitching of continuity (5). The risk is drifting into vigilance: endless micro-tweaks to stay “*authentic*” across shifting contexts, a never-finished curation loop where continuity is maintained but anxiously so (50). Across HPE, digital identity is described as fluid and dynamic, socialized within communities of practice and tracked by platform signals (e.g., followers/traffic). Attempts to separate personal/professional streams often converge in practice; when online and offline selves misalign, participants report identity conflict and active management to re-stitch coherence; a classic cautious mediation under visibility pressure (26, 51). Wang et al. (52) demonstrate “*outsourced meaning-making*,” where viewers infer a user’s persona from recent posts across key personal characteristics, and their inferences diverge from self-reports. Multimedia posts raise accuracy but lower consensus, while text yields higher consensus, suggesting that identity online is co-authored by a transactive cyber-audience rather than the speaker alone. In that inherently noisy loop, anxious vigilance, our mediating “*digital neurotic self*”’ reads as an *adaptive* response to an audience that both completes and distorts the message. In the midst of all this, the digital neurotic self echoed as follows:

“*Contemporary identity*” *connected to* “*unlimited supply*” *intrigues me.*

* I think about the infinity mirror effect.*

* Sherman and I are both* “*self-taught.*” *We distrust social media images.*

* I reflect again upon the infinity mirror.*

* I continue searching online* (53).

### 3.2 Trope II: instrumentalised mode — the “*digital psychotic self*” (metaphorical)

The second, more precarious figure is a digital psychotic self - a presentation built for consumption that drifts from an authored narrative and aligns identity with algorithmic gratification (44). Because the digital world makes identity editable and performative, visibility cues such as likes, shares, and notifications pull presentation toward what appears legible, privileging consumable tropes over nuance (1). In this mode, the situational self overwhelms the substantive self; counters and formats eclipse values and tune performance to platform legibility (28). The insignia manifests itself as a response to the constraints and laws of the Symbolic order (7). In this sense, the Real holds the prospect of transgressing the Symbolic in pursuit of a fuller, more “*original*” desire (7). This recognition is tied to the desire of the Others. Individuals seek validation from significant others: parents, peers, and society, which shapes their behavior and sense of self - “*the desire to be desired.*” Technology is rendering the other’s response increasingly unstable. At the same time, this “*commercial surveillance*” produces a psychic numbing that inures us to the realities of being tracked, parsed, mined, and modified, eventually “*leaving us singing in our chains*,” a drift from exploration toward instrumentalized self-construction tuned to platform legibility (54). In Bishop and Kant’s (54) workshop materials, “*algorithmic autobiography*” reads as a beautiful narration of surrender to platform materiality, identity co-authored by ad-interest taxonomies and interface logics. These creative outputs show how profiles, categories, and ad interests pre-format self-stories; the resulting “*algorithmic autobiography*” *feels* authored yet is steered by infrastructures, hence precipitating this psychotic vicious cycle.

*I am depicted as mindless, unfeeling and vain.*

* Consuming luxury, devouring fame.*

* I am empty and superficial.*

* Stocking up on weight loss drugs and shame*

* I am well-dressed but not well liked.*

* I only have the Facebook algorithm (and myself) to blame* (54).

Holmes (42) shows how the noise of the network bleeds into ordinary perception; identity becomes interwoven with technical assemblages, reading here as instrumentalised selfhood co-authored by infrastructures and metrics. Self-presentation is optimized for legibility and rewards (likes, attractiveness cues) exemplifies identity tuned to counters rather than meaning (5), leading to a false self-presentation that departs from one’s genuine character and values. However, it’s not always deception but does include experimentation of idealized projection (filters, transformed self, perfected traits), making it “*a more defensive, protective self that hides one’s* “*true self*”.” Peer-level *injunctive norms* (what others expect you should do) and broader social norms often drive this, and the self is tuned for approval and popularity. A Q-methodology study reframes the “narcissist selfie” trope into four shared understandings; “*Presenting*… *Me!*”, “*I am what I am*,” “*Sharing is caring*,” and “*The in-crowd: beautiful and popular*” with the first and fourth most clearly aligned to instrumentalised, approval-optimized performance (55). In effect, these approval-optimized routines open a path to micro-celebrity, where attention becomes the organizing value and anyone can be staged as a persona at scale (33, 56).

Micro-celebrity culture normalizes performance for attention. Personas are tailored to trends, engagement metrics, and audience appeal, and platform-coded cultural capital; looks, tropes, timing, and vernacular convert directly into visibility (33, 56). Unlike broadcast media, social media makes users creators, critics, and carriers of content, generating a dense, searchable trail of first-person opinion; much of what circulates reads as felt experience even as it is shaped by affordances and metrics. The glass-walled nature of these spaces exposes college students’ psychological development and self-perception to constant feedback, with effects on social integration, self-esteem, mental health, and academic performance (57).

The empirical pattern is consistent across studies of students and trainees (5). Social media’s glass-walled affordances expose psychological development and self-perception to constant feedback; effects are mixed, linking visibility work to both perceived autonomy and happiness, and to anxiety, depression, loneliness, and achievement pressure (5, 55, 57). Continuity thins as archives, trends, and engagement norms overwrite trajectories; learners, clinicians, and academics are nudged toward narrow, high-yield selves; the always-confident educator, the endlessly productive scholar, the patient-pleasing clinician shaped by rating sites. Agency becomes co-authored with interfaces: people chase formats and counters, self-censor under moderation shadows, and accept default taxonomies of who counts and how. In HPE, this instrumentalisation appears when e-portfolios function as performance dashboards, when altmetrics and patient-rating websites discipline presentation, and when novices feel compelled to make small wins permanent for institutional visibility (34). Cass’s (58) cinematic analogy captures the feel: carefully curated images of an idealized self against perfect backdrops with flawless lighting and angles; experience is paused and posed so a consumable “*movie-life*” can circulate. In Goffman’s (59) terms, they craft what they think the audience will find pleasant and acceptable, and the counters rewards in the modern William H. Vanderbilt’s American Horse Exchange Times Square – *the digital media*.

## 4 Discussion

This discussion returns to the guiding question of the review: how digital platforms reshape, constrain, and co-author identity and interprets the results through the same symbolic interactionist and sociomaterial lenses that structured our analysis. First, digital identity is now a primary site where the self is performed, negotiated, and sustained. Bringing symbolic interactionism (co-constructed selves, impression management) and sociomaterial theory (platforms/artifacts co-produce action) into one frame (29, 30), we treat digital identity as both symbolic performance and material enactment. Read through the lenses of authenticity, visibility, continuity (idem/ipse), and agency, our corpus suggests two recurrent modes: a mediating “*digital neurotic self*” (authorship under constraint) and an instrumentalised “*digital psychotic self*” (presentation tuned to platform legibility). Empirically, affordances and metrics co-author identity work; homophily drifts toward community sameness; and narrative coherence frays unless people actively curate via audience design, selective disclosure, and narrative stitching. In HPE, where online presence is part of professional identity formation, we encourage treating digital identity as a dimension of that formation; coach learners and faculty in authorship and stewardship (audience design, narrative stitching, platform literacy) rather than only compliance, so convergence is handled deliberately and misalignment reduced (26, 43).

Together, these findings illuminate the core tension at the heart of our research question: the struggle to sustain authored identity (ipse) under sociomaterial pressures that pull identity toward legible, platform-shaped sameness (idem). We now step back from the results to make sense of what they add up to. Reading the corpus through symbolic interactionism and a sociomaterial stance, we first articulate the problem: how platform logics and overlapping publics reshape identity work in HPE, then propose strategies to reclaim authorship and agency, and finally acknowledge the limits and situatedness of our argument.

### 4.1 Problem identification

In line with the results, we circle back to scholarship that often splits between psychosocial expression and platform-centric algorithmics. Our synthesis brings symbolic interactionism and sociomaterial theory together to illuminate digital identity as both symbolic performance and material enactment across these domains (29, 30). Historically, John Locke and Immanuel Kant’s self-sameness (36); the conviction that one’s identity remains stable across time and situation was foundational, grounded in an intact thread of memory. However, Kant’s ontological assumptions that self-sameness is the nidus around which we weave our roles and actions, even in the unexpected undulating transitions, are being challenged in the collapsing contexts of the digital world. The assumption is strained by context collapse. We are no longer engaging with a single audience, but presenting ourselves simultaneously to multiple and often conflicting communities, shattering the fourth wall (60) and disrupting the notion of stability (61) giving rise to “identity diffusion” (62) - a fluid, performable self that is also fragmented and dispersed across platforms and audiences; many users lack a coherent life story of the “*self*” others encounter online (43).

Read through an ipse/idem lens, we see community sameness crowding out authored uniqueness. Large-scale analyses of 85,000 Twitter users show overlapping self-labels/hashtags pulling people into clusters with prototypical tags; as clusters grow, vocabularies become less conspicuous, raising idem-sameness and thinning the ipse-authored selfhood, a collective legibility that flattens idiosyncrasy (63). A sociomaterial view clarifies why this is not only a social expectation but a mattering of the matter - algorithms, metrics, and templates rewarding what is legible and ignoring what is not. Over time, professional identities risk becoming algorithmically curated; neat, polished, uniform, trading quirks and tensions for the comfort of visible, performant norms (10). We are not self-authored, rather platform-dictated.

Quantification accelerates the drift (63). Likes, shares, followers, and comments atomize identity into countable units that do not merely reflect popularity but shape behavior (3, 4). We become attuned to produce clickable, likeable, shareable, and relatable content. This is exactly what sociomaterial theory reminds us of. These technologies are not inert. They coerce us. They nudge and exploit us. And we are being so pliable to an extent that we have reduced our personas to ostensible fragments to be consumed, evaluated, and rewarded by others, externalizing our self-efficacy (46). As reported in our results, this reconstruction can be exploratory and performative, pressures from audience, metrics, and privacy can either hold users in perpetual moratorium (exploring without committing) or pull them into platform-legible sameness (5).

To be clear, we are not claiming that digital expression is fake or unreal. The sharper question is whose intentions it carries; our own or the algorithmic gaze (64)? With Ricoeur (65), idem and ipse co-constitute the narrative self, yet in the digital age that narrative is increasingly scripted by external signals, leaving less room for dissonance, reflection, and musing. While the narrative self was once a harbinger of coherence across shifting contexts, it now risks becoming a patchwork of algorithmically tuned impressions, shaped less by meaning and more by performance, putting an impression of me that doesn’t reflect who we are. One may rightly argue that identity is a contextual product of relational and cultural forces, constructed socially and has never been fixed or formed in isolation (65). In today’s world, many of these relational forces reside in digital spaces, making social media a primary site for identity construction. Hence, the digital identities may well feel real to those who inhabit them. However, the question then becomes not whether these identities are organic, but rather how much agency individuals retain in shaping them, and at what cost. Also, we don’t hesitate to agree that digital spaces were meant to imitate real social environments for many purposes and have successfully catalyzed new ways of being (1). Today’s inhabitants are turning to online platforms to explore emerging identities and acquire language that helps them articulate who they are becoming, which may not yet find space in their offline lives (66). However, this exploration may reflect aspirational or idealized versions of the self.

From a psychoanalytic perspective, one might suggest, that such expressions emerge from the dynamics of the self. The self, which is shaped by the Id (human’s basic instinctual drives), the Ego (reality principle), or the Superego (values and morals of society) (67). In this case, the digital identities, even when performative, can be purposeful articulations of growth or change. The challenge arises not from identity shifts themselves, but when these shifts are too heavily shaped by metrics, performance expectations, or external validation, rather than conscious, intentional self-authorship (10). These sociomaterial conditions do not merely influence identity conceptually or socially, but they also manifest psychologically.

### 4.2 Strategies to overcome the problem

Reclaiming authenticity – “*becoming the real*” - asks us to make conscious decisions, avoid impulse publishing, resist affirmation by metrics, and write stories that reflect our true aspirations and values rather than succumbing to curated sameness. You can do this by bringing back the narrative self, embracing agency, moving beyond continuous exploration, and prioritizing self-perception. Each move helps navigate the tension between fitting in and standing out, building a more durable online presence.

#### 4.2.1 Revive the narrative (story) self

As Ricoeur (65), Foucault (68) remind us, stories are more than self-expression; they are a way of knowing, becoming, and resisting. Life stories hold many parts and yield psychological understanding (69). Alas, storytelling is hijacked by imagined online audiences. We craft posts to do well with algorithms rather than to make sense. To avoid this, we need to take storytelling back as a personal, situational act aimed at clarity and purpose, not the counter. Even if the self is scattered across platforms, it can be intentionally assembled into an “*authentic rosary*” by linking posts, interactions, and impressions. Echoing the findings of Claudia Mellado’s ethnographic study (70) where Chilean journalists identities are performed through service, celebrity (33), promoter, and joker roles, HCPs can storyboard a professional narrative by naming the protagonist (clinician-educator/researcher), clarifying what the story wants to convey, setting the audience and context, and choosing the props (artifacts/evidence) and the syntax/voice per platform. Hence, continuity holds across channels.

#### 4.2.2 Return to self-perception as resistance

In gaze-saturated environments, the first-person perspective, the ability to see, feel, and author oneself diffuses. A simple inward pause, *Who am I? Why am I?* can disrupt the performative script (18). These questions re-center identity from impression to intention. Drawing on small-stories work by McGannon, Bundon (71), we treat brief, first-person vignettes as a practical way to loosen platform-driven sameness: narrate what you do, value, and learn so an authored *ipse* can thread through spaces that rewards conformity. For HPE, this means brief, situated stories that let difference show while staying legible, turning visibility into a record of work rather than a performance for metrics. When platform signals and audience tastes shift by the hour, the “Other” no longer offers a stable answer to what one should want; that instability opens a pedagogic space to practice “*Che vuoi?*” (*What do I want?)* as Lacan puts it, and to anchor choices in authored values rather than fluctuating counters (7).

#### 4.2.3 Restore backstage and reciprocity; don’t let context collapse run the show

Goffman’s dramaturgy reminds us that the self is staged across frontstage and backstage; in digital life, the backstage recedes as the ambient, algorithmic gaze collapses contexts into a single, public-facing performance, pressurizing the Eriksonian work of coherence across time, messing with the reciprocal impressionism which sits at the heart of symbolic interactionism (63). And then Foucault’s panopticon reminds us, the mere possibility of being watched reshapes conduct; on social media, that imagined, potentially limitless audience produces what Marwick (33) calls “lifestreaming,” seeing oneself through others’ gaze and continually editing behavior to sustain a desired self-presentation. Profiles and posts become strategic broadcasts rather than sites of relational disclosure, feeding inter-community sameness (57). To regain authenticity, reclaim reciprocity, decide when and where to share, refuse the need always to be seen, and treat audiences not as consumers but as co-narrators.

#### 4.2.4 Practice agency as quiet refusal; teach the languages of platforms

At the heart of this resistance lies agency. Following Emirbayer and Mische (72), agency is the reflexive structuring of action across past, present, and future. In sociomaterial environments where architectures shape both content and visibility, agency emerges as small, stubborn moves: pausing before posting; publishing less performatively; prioritizing meaning over metrics —a subtle yet radical form of self-authorship. One element that strengthens agency is sociolinguistic competence. In the Kazakh study, bloggers make their mark through pragmatic moves of code-switching and hybrid registers that signal cultural, social, and professional positioning; for HPE, this argues for teaching platform sociolinguistic competence (choosing register/code and multimodal cues to fit audience, goal, and role) (73). Empowerment rises when people can author identity on their own terms (choice of spaces, audience control, community support). Disempowerment rises when platform rules, counters, and moderation discipline legibility so pair pedagogy with platform literacy and safety practices (privacy, pseudonymity, small publics) and press for technical/organizational fixes (better security, fair moderation) since outcomes hinge on both people and infrastructure (74). The practical implication is clear: de-center counters, teach how feeds rank and normalize, and provide counter-spaces; low-exposure, feedback-rich venues so identity develops as authored work rather than as an artifact of the algorithmic gaze (33).

#### 4.2.5 Move beyond perpetual exploration; commit, mirror, and align the *idem* and *ipse*

Second accounts can be safe sites for trying things on (2), but perpetual exploration easily becomes an endless loop (62, 75). In professional and developmental contexts, clarity and direction matter. Make conscious commitments to values, roles, and relationships that extend beyond platforms. Practically, *mirror* your web presence (45) by a periodic audit and aligning the scattered traces (17), pruning the stale, and tuning who sees what, where, and when. Keep the *idem* (what others assemble) roughly in step with the *ipse* (what you mean to author). This is the neurotic labor of continuity—small, regular corrections under a platform gaze. We encourage you to stop staging a “movie-life” and return to unselfconscious doing, where essence leaks out in the ordinary (58). HCP hiring and promotion now read the digital self alongside the CV. Committees and patients will Google you. They skim page-one results, LinkedIn, Google Scholar/ORCID, and even patient ratings, looking for a coherent, value-anchored story (substantive self) aligned with the situational self and for the red flags (boundary breaches, tone, misinformation) (17). Structured pedagogy can convert exploration into commitment. In an RCT, a digital program pairing reflection with prescribed social-media activities (targeted information-seeking and connection) increased exploration and commitment; greater engagement was linked to lower ruminative exploration (6).

#### 4.2.6 Assume the web is public; build literacy and better rooms

Even in “chiaroscuro” corners, anything can surface to unintended audiences. This isn’t self-censorship; it’s fit-for-audience judgment. Before posting, run a quick scan: “why this, for whom (now and later), and will I own this trace?” The same socio-technical systems that splinter identity can be repurposed as reflective tools, when traces are remediated (e.g., into a book, triptych, or film), people reduce noise, surface narratives, and archival values re-stitching continuity, indicating that social media can also enable self-reflection by design (43). Treat platforms as a diasporic resource; they enable boundary-crossing, political voice, and reflective self-making, but credibility and audience design must be taught (family/WhatsApp circuits vs. peer networks). Teach credibility, audience choice, fact-checking, and small-stakes narrative practice (39). For HPE, teach identity-congruent sharing (stay close to domain values; curate coherence) and make audience/metric effects explicit (as audiences grow, posts skew toward what “fits” that identity); pair this with periodic audits to avoid dispersive broadcasting and echo-chamber homophily (27, 38, 39). Curricula should build connectedness capabilities—social-network literacy, purposeful audience building, and e-portfolio practice, so identity work moves from “assignment pieces” to value-anchored, outward-facing presence (51).

### 4.3 Limitations

Moreover, we acknowledge that our argument may risk overgeneralisation as identity is deeply contextual. Variables such as age, life stage, and cultural orientation significantly shape how individuals perceive and present themselves online (66). For example, someone still forming a stable sense of self may engage with social media differently than someone reflecting on a life already lived. Evidence with Spanish adolescents shows self-presentation is pulled by several forces at once: authenticity, social approval, and image control and, for many, likes/followers were not the main driver; practices also shifted with age, gender, and the platform’s affordances (e.g., filters vs. video) (76). Those from collectivist orientations believing in interdependent self-construals may prioritize digital harmony, favoring community sameness. Individualists, who rely on independent orientations, may prioritize individual expression (66). These distinctions shape whether digital identity feels coherent or fragmented (9, 10). However, across these contexts, the common thread remains that sociomaterial environments now play a dominant role in directing identity performances. Whether subtle or overt, the pressures of algorithmic legibility pervade, and unless we critically examine this shaping force, we risk mistaking performance for authenticity and recognition for selfhood. We recognize our critique may read as a *Lord of the Flies* portrayal in which platforms mint only narcissists; our intent is to surface structural pressures on identity work, not to deny the countervailing evidence of care, creativity, and agency.

Before concluding, we acknowledge that the synthesis provided is not exhaustive, as this was never the intent of our write-up. Additionally, examples in this article come from the experiences of health professionals and medical students; however, the dynamics discussed here are not limited to clinical education or the healthcare sector. The way authenticity, performance expectations, and platform logics interact is a part of our modern life that affects how identities are created, curated, and consumed in all kinds of professions, cultures, and public spaces. This analysis focuses on the sociomaterial and symbolic aspects of digital selfhood. It builds on the work of empirical and non-empirical research in a wide range of fields, including media studies, education, organizational research, and cultural sociology, to offer new ways to think about identity work in environments that use technology. Our write up encourages new ways of knowing and doing things, making us reassess where identity work happens, and how we could deal with the material-discursive entanglements that make up the self in networked settings.

### 4.4 Conclusion

In conclusion, getting back control of our stories in the digital age does not mean shunning social media or wishing for a return to so-called “real” life. Instead, it is about making a conscious effort to be consistent and creating digital identities that reflect who we are and what we believe, rather than just what we measure. We have used sociomaterial theory to say that identity is no longer just shaped by people and social conventions, but also by the algorithmic and material features of digital platforms. These platforms are not fair and neutral. They quietly whisper what should be shown, seen, valued, and reinforced. In such settings, reclaiming authorship requires deliberate and intentional acts of reflection. Simple practices of private contemplation before public expression, digital journaling to track storyline, or even periodic self-checks asking “*Is this aligned with who I am becoming?*” and restore intentionality. These are not rigid prescriptions but invitations to pause, to write, to notice, to reconnect. As the fragments of the self continue to scatter across feeds, stories, and scrolls, these moments of reflection allow us to thread them back together in a beautiful rosary. They allow us to become visible not merely as content, but as selves in motion; complex, situated, and still becoming.

As we reflect on the interplay of digital pressures and self-expression, reclaiming narrative agency is not only a personal act. It is a sociomaterial intervention, a refusal to let platforms fully choreograph the becoming of the self. Let’s strive to be more than visible. Let’s be real. Let us not wait until we feel like strangers in our own lives, echoing the words of Edgar Allan Poe;

*From childhood’s hour I have not been*

* As others were - I have not seen*

* As others saw - I could not bring*

* My passions from a common spring*

* From the same source, I have not taken*

* My sorrow - I could not awaken*

* My heart to joy at the same tone*

* And all I lov’d - I lov’d alone*
