# Correction: When the self “*logs in”*- a critical narrative review of digital identity in health professions education

**DOI:** 10.3389/fmed.2025.1770708

**Published:** 2025-12-23

**Authors:** Frontiers Production Office

**Affiliations:** Frontiers Media SA, Lausanne, Switzerland

**Keywords:** digital identity, digital platforms, socio-material theory, impression management, narrative self, digital agency, health professions education, symbolic interactiosnism

Affiliation “College of Medicine, Gulf Medical University, Ajman, United Arab Emirates” was erroneously given as “College of Medicine, University of Sharjah, Sharjah, United Arab Emirates”.

An abbreviation error was identified, where the full abbreviation for “healthcare professionals” was not included. A correction has been made to the Section 1. **Introduction**, Paragraph 3. The sentence was: “For healthcare professionals (H), identity work increasingly unfolds in public, persistent, searchable spaces where platform logics shape what is sayable and seen (11)”. The sentence has been corrected to: “For healthcare professionals (HCPs), identity work increasingly unfolds in public, persistent, searchable spaces where platform logics shape what is sayable and seen (11)”.

The original version of this article has been updated.

